# Subclinical Hypothyroidism Affects Postoperative Outcome of Patients Undergoing Total Knee Arthroplasty

**DOI:** 10.1111/os.12934

**Published:** 2021-04-04

**Authors:** Wen Jing, Gong Long, Zhao Yan, Yi Ping, Tan Mingsheng

**Affiliations:** ^1^ Department of Endocrinology and Metabolism Shanxi Bethune Hospital, Shanxi Academy of Medical Sciences No. 99, Longcheng Street Taiyuan City Shanxi Province 030032 China; ^2^ Department of Orthopedic, China‐Japan Friendship Hospital China‐Japan Friendship Hospital, Peking Union Medica College, Chinese Academy of Medical College No.2 Yin Hua East Street Beijing 100029 China; ^3^ Department of Orthopaedic Surgery the 980th Hospital of Joint Logistic Support Force of PLA. Shijiazhuang He Bei Province 050000 China

**Keywords:** Clinical outcomes, Comparison, Subclinical hypothyroidism, Total knee arthroplasty

## Abstract

**Objective:**

The aim of this study was to investigate whether subclinical hypothyroidism could increase the risk of postoperative complications in patients undergoing primary total knee arthroplasty (TKA).

**Methods:**

A prospective case‐control study of 796 patients undergoing primary TKA between January 2015 and January 2020 was performed. A total of 700 patients (87.9%) were female and the average age of included patients was 65.0 years, with a standard deviation of 5.6. The participants who had subclinical hypothyroidism were referred to as the case group, while those without abnormal thyrotropin (TSH) were included in the control group (matched for age and gender). The fasting plasma levels of TSH were tested in the morning in all patients. The diagnosis of subclinical hypothyroidism was completed by a senior endocrinologist based on laboratory tests; namely, a serum TSH ≥ 5 mu/L and normal free thyroxine (FT4). Subclinical hypothyroidism was further described as mild (TSH < 10 mu/L) or severe (TSH ≥ 10 mu/L). The incidence of 90‐day postoperative complications was compared between two cohorts. Logistic regression analysis was used for the risk factors of 90‐day postoperative complications following TKA.

**Results:**

A total of 398 patients had a diagnosis of subclinical hypothyroidism. Among them, 275 cases (69.1%) were described as mild (79 patients [19.8%] with low FT4 and 196 patients [49.2%] with normal FT4 in the repeated test) and 123 cases (30.9%) as severe subclinical hypothyroidism. Of the 196 patients (49.2%) with mild subclinical hypothyroidism and normal FT4, 63 patients (15.8%) had symptoms before surgery. Patients were followed up for an average duration of 25.4 months (6 to 43 months). A total of 265 patients (66.6%) received preoperative treatment for subclinical hypothyroidism, with an average therapy time of 9.2 months. There were 162 patients (40.7%) with positive autoantibodies to thyroid peroxidase (anti‐TPO). There were no statistically significant differences in baseline data between cohorts (all *P* > 0.05). As for the cumulative 90‐day outcomes, subclinical hypothyroidism increased the incidences of both medical and surgical complications following primary TKA compared to those without this condition (11.6% *vs* 7.2%, OR = 1.55, 95% confidence interval [CI] = 1.47–1.62, *P* < 0.05). Subclinical hypothyroidism caused patients to suffer increased total incidence of readmission within the first 90 days after discharge when compared to those without this condition (20.61% *vs* 14.15%, OR = 1.45, 95% CI = 1.41–1.49, *P* < 0.001). Controlling for preoperative and intraoperative variables, the patients with TSH ≥ 10 mu/L and positive anti‐TPO and those without corrected subclinical hypothyroid and thyroid hormone supplementation were more likely to experience postoperative complications within 90 days of TKA.

**Conclusion:**

Subclinical hypothyroidism might increase the risk of postoperative complications within 90 days of TKA, especially for the patients with TSH ≥ 10 mu/L and positive anti‐TPO and those without corrected subclinical hypothyroid and thyroid hormone supplementation.

## Graphical abstract

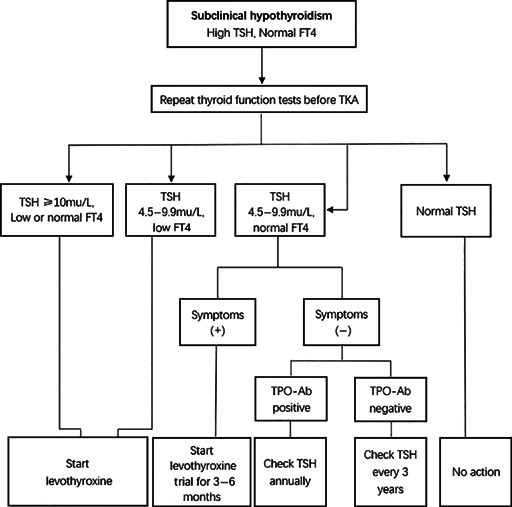

## Introduction

Total knee arthroplasty (TKA) is one of the most clinically cost‐effective and successful treatments for severe knee osteoarthritis (KOA) and significantly improves the quality of life (QoL) of patients with this condition^1^, ^2^. In the United States, more than 500,000 TKA are performed annually, and if the present trends continue, more than 3.5 million TKA will be performed each year within the next 25 years^1^. A satisfactory result after TKA involves various elements, such as thorough surgical planning before surgery, strict selection for both patient and prosthesis, surgical technique, pain management, and functional exercise. As the utilization and cost of TKA grow, many stakeholders are increasingly concerned about the investigation and identification of modifiable variables, which might allow for intervention before TKA, and this could potentially contribute to good clinical results and relieve the heavy economic burden on the healthcare system^3^. Surgical planning ought to identify potential factors related to adverse events following the surgery and avoid them as much as possible^4^. Commonly known risk factors include increasing age, obesity, smoking, low nutritional status, as well as endocrine disorders^5^. Some endocrine dysfunctions, such as thyroid disorders and diabetes, play a vital role in the occurrence of postoperative complications^6^, ^7^.

Thyroid hormones are of great necessity to the growth and development of every tissue and organ because they are extensively involved in the regulation of the daily process of metabolism of almost every cell^8^. Hence, thyroid dysfunction can compromise recovery and have severe consequences for patients following surgery^7^. For example, Tan *et al*. demonstrated that hypothyroidism increased the likelihood of periprosthetic joint infection (PJI) in a retrospective case‐control study^9^. Another clinical study based on a larger population revealed that hypothyroidism could lead to an increased 90‐day incidence of multiple postoperative complications, such as infection and acute postoperative anemia, and higher medical costs for patients following the TKA^10^.

Subclinical hypothyroidism, representing more subtle abnormalities in thyroid function, is considerably more common in comparison with hypothyroidism^8^. Unfortunately, this condition does not receive much attention in the orthopaedic field. Subclinical hypothyroidism is also referred to as mild hypothyroidism, or compensated hypothyroidism. This condition is diagnosed as an elevated thyroid‐stimulating hormone (TSH) level with serum‐free thyroxine (FT4) in the normal range^11^. Of note, in subclinical hypothyroidism, thyroid hormone levels in the blood remain in the normal range. However, the real levels are slightly lower than they should be for that particular individual. In most cases, the condition is so mild that a person might fail to feel they are experiencing it, as the symptoms frequently accompanied by hypothyroidism are absent.

Subclinical hypothyroidism is a common disorder, particularly for older women. It is reported to occur in nearly 10% of older women^11^. The prevalence is obviously higher in those of advanced age, and it is reported that up to 20% of women aged 75 years or above have elevated TSH levels^11^, ^12^. A previous study demonstrated that when an individual's serum TSH levels continue to rise, thyroid hormone levels are no longer at a normal level for that person^13^. Based on what we know about the physiological process of pituitary–thyroid feedback at present, persons without TSH levels that are not in the normal range experience minor pathological changes in thyroid function from their previously genetically‐determined set point. Compensatory elevation in TSH levels occurs with these changes, which reinstates the normal thyroid function to some extent^7^, ^8^. Isolated abnormal TSH levels may be a transient phenomenon with no significant consequences. Unfortunately, in many people, abnormal TSH levels can persist for a rather long time until the condition develops into overt thyroid disorder over time^8^.

Despite subclinical hypothyroidism being accompanied by mild thyroid dysfunction, it is still a clinically meaningful disorder, especially for the elderly. There are adverse effects, at least in some individuals, that require treatment^11^, ^13^, ^14^. The effects of subclinical hypothyroidism and the benefits of its treatment on health, especially in the elderly, have been debated for several decades. Some studies have shown that long‐term complications of subclinical hypothyroidism without clinical intervention involve cardiovascular disease, cerebrovascular disease, cognitive impairment, low metabolic parameters, osteoporotic fractures, and mental health problems, which are not conducive to the recovery after TKA^9^, ^11^. However, there is no substantial evidence that levothyroxine treatment can help reduce the adverse events mentioned above. In addition, most individuals with subclinical hypothyroidism have no symptoms. Thus, the ideal treatment method remains controversial^11^.

Due to the high frequency and severity of this condition, essential questions have been raised regarding its clinical relevance and appropriate management in the field of arthroplasty. Therefore, the aims of this clinical research were to (i) determine the impact of subclinical hypothyroidism on postoperative complications among patients accepting primary TKA; (ii) understand which subclinical hypothyroidism patients suffered increased risks of 90‐day complications after TKA; and (iii) provide preoperative management recommendations for subclinical hypothyroidism to help reduce these potential risks and to decrease the medical costs in these patients. To our knowledge, this is the first study to evaluate whether subclinical hypothyroidism could increase the likelihood of medical complications following TKA.

## Methods and Materials

### Study Design and Study Population

Inclusion criteria for the case group included (i) diagnosis of osteoarthritis and subclinical hypothyroidism following the International Classification of Diseases 9th Revision (ICD‐9); (ii) undergoing unilateral and primary TKA; (iii) having complete data, including for clinical evaluations and examinations, for comparison; and (vi) a prospective study.

The exclusion criteria were: (i) a diagnosis of hyperthyroidism (ICD‐9 codes 242.0–242.3 and 242.9) and hypothyroidism (ICD‐9 codes 2440–2443 and 2448); (ii) an isolated TSH elevation such as mild thyroid gland failure (the hypothalamic–pituitary–thyroid–peripheral tissue axis must be intact); and (iii) other causes of TSH elevations, such as nonthyroidal illnesses or medications^15^, ^16^.

Following institutional review board approval at our hospital, a prospective, case‐control study of 796 patients undergoing primary TKA between January 2015 and January 2020 was performed. All included subjects provided written consent before the start of the study. The patients with subclinical hypothyroidism were selected as the case group and those without abnormal TSH as the control group.

After the establishment of the cohort, two cohorts with the same distribution for age and gender were randomly selected. Whether subclinical hypothyroidism presented or not acted as the sole differentiating factor. Prior to the analysis, patients were matched in a 1:1 ratio based on age, gender, and body mass index (BMI).

### Testing and Diagnosis for Subclinical Hypothyroidism

Subclinical hypothyroidism was further described as mild (TSH < 10 mu/L) or severe (TSH ≥ 10 mu/L)^11^. Diagnosis of subclinical hypothyroidism is based on laboratory examination, completed by a senior endocrinologist.

The fasting plasma levels of TSH were tested in the morning in all patients. When the serum TSH level was higher than the normal laboratory reference range (TSH ≥ 5 mu/L) and serum‐free T4 (FT4) was within the reference range, it met the diagnostic criteria^11^. TSH and FT4 levels should be evaluated once again within 2–12 weeks to exclude the influence of laboratory errors or temporary elevation^16^.

Subclinical hypothyroidism is confirmed with further evaluations involving symptoms and signs, previous treatment (such as radiotherapy, drugs, and partial thyroidectomy), goiter, and family history of thyroid disorder.

### Surgery Process

All TKA were conducted by the same surgical team in their respective institutions with epidural or general anesthesia. For the exposure of the knee, an anterior midline skin incision and a medial parapatellar approach were applied. A measured resection technique was used to address the extension and flexion gap. A distal femoral cut was performed with an intramedullary instrumentation setting of 6° of the anatomic valgus. Referring to the surgical transepicondylar axis, the femoral external rotation cut was performed. Extramedullary instrumentation was used to achieve a target tibial cut of 90° relative to the mechanical axis in the coronal plane and of 3° to 5° relative to the posterior slope in the sagittal plane.

Cementless as well as cruciate‐retaining prostheses (Gemini MK‐II, Link, Germany) with patellar resurfacing were used. They are suitable for joint space in the absence of severe knee deformity (flexion contracture and varus deformity less than 15°) and with an intact posterior cruciate ligament

The drainage was removed according to the drainage volume within 24 h after surgery. The tourniquet devices were set at approximately 55 kPa–75 kPa for around 40 min. Patients were given cefuroxime prophylaxis 30 min before the operation and within 24 h after the procedure (1.5 g, iv, tid). Prophylactic anticoagulant therapy (rivaroxaban or low molecular weight heparin) was started within 12 h after the operation and lasted for at least 28 days. We encouraged patients to start weight‐bearing as soon as possible (generally within the first 24 h) with the help of walking‐assistance devices. Whether physiotherapists were needed depended on the functional performance of that particular patient. The X‐ray films of all the patients showed that the alignment of the lower limbs in the weight‐bearing position and the non‐weight‐bearing position was good, with sound component placement.

### Sample Size

G Power 3.1.9.2 was used to calculate the power of the study, with an effect size of 0.3, the first error of 0.05. As a result, the present study included 231 patients undergoing TKA. The calculated study power is good, with a result of 94.25%.

### Nursing and Treatment Protocol

**Fig 1:**
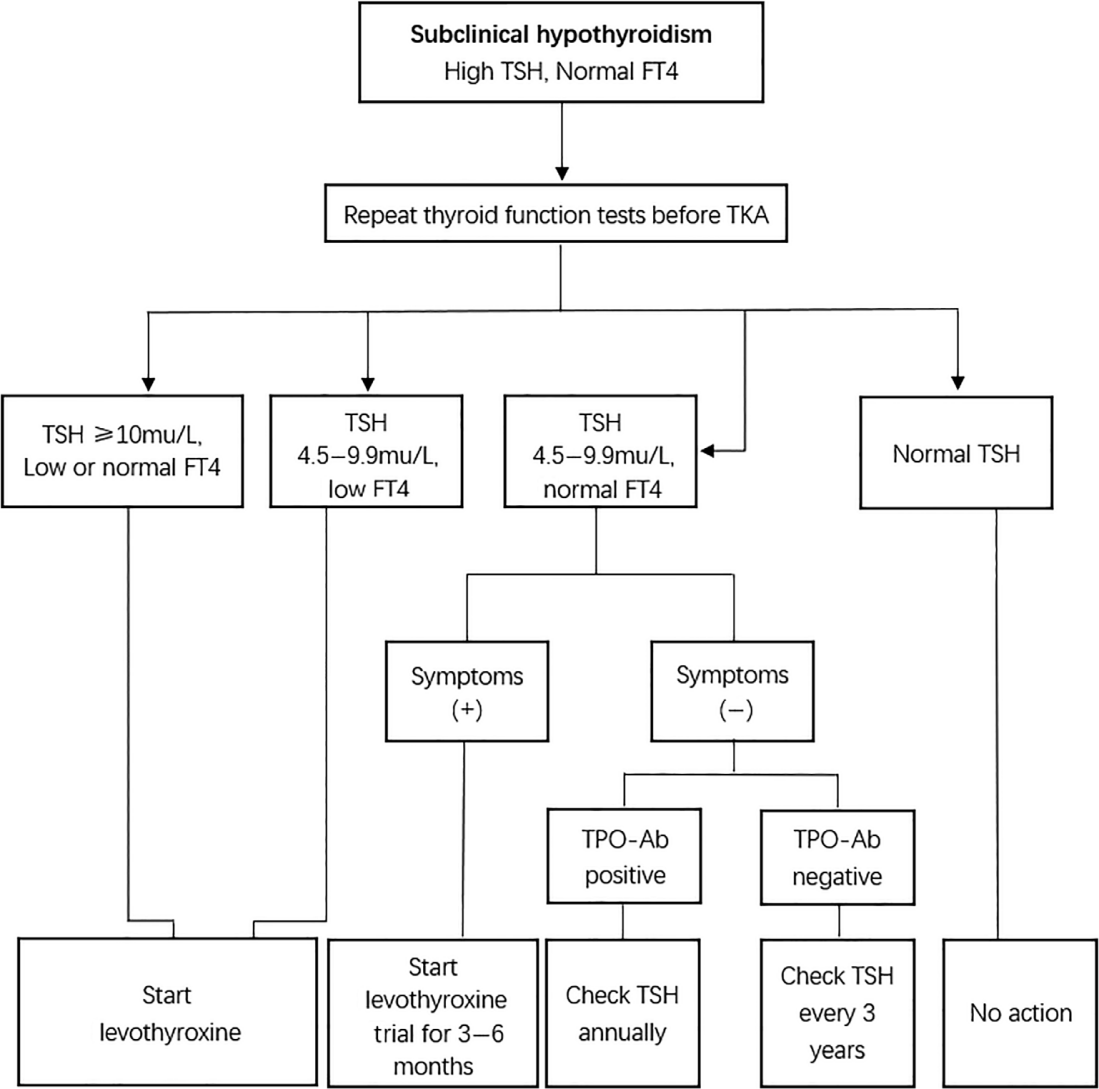
Flow chart for management of subclinical hypothyroidism in the candidates for total knee arthroplasty (TKA). FT4, serum‐free thyroxine; TPO‐Ab, autoantibodies to thyroid peroxidase; TSH, abnormal thyrotropin.

The treatment of subclinical hypothyroidism is based on guidelines by the American Endocrine Association (ATA) (Fig. 1)^17^, ^18^. Symptoms and signs of hypothyroidism ought to be evaluated at each follow up following the initiation of the administration of the thyroid hormone. The symptoms of thyroid hormone deficiency can be effectively alleviated in several weeks to months in most cases by treatment with levothyroxine (LT4). The dose needs to be monitored by evaluating serum TSH at least 2 months after the start of treatment. It was not adjusted immediately if small alterations from the targeted value of TSH occurred. The TSH test can be repeated after 2–3 months prior to determining the dose change. Serum TSH was measured after 6 months if it reached a normal range. Annual monitoring was required if a stable LT4 dose was obtained.

In many cases, subclinical hypothyroidism in elderly patients is characterized by a paucity of specific signs and symptoms. The symptoms may be subtle and include hoarseness, deafness, confusion, dementia, ataxia, depression, dry skin, and hair loss. At the preoperative stage, LT4 should be administered in a titrated manner to normalize the thyroid function. A normal starting dosage of LT4 in an elderly subject is approximately 1 mg/kg/day, which is maintained for 4–6 weeks^19^. If heart disease is suspected, a lower starting dose is appropriate. Dosage adjustments are guided by the response in TSH and the clinical state, with emphasis on possible cardiac adverse effects. Limited evidence suggests that treatment of subclinical hypothyroidism with serum TSH of up to 10 mIU/L should probably be avoided in patients older than 85 years because subclinical hypothyroidism in this subpopulation does not result in adverse effects and is, instead, found to be associated with prolonged life span^20^, ^21^.

No or subtle specific signs and symptoms can be detected in many patients. Symptoms include hoarseness, deafness, delirium, dementia, ataxia, depression, dry skin, and hair loss. The standard initial dose of LT4 was approximately 1 mg/kg/day for 4–6 weeks^19^. The initial dose should be reduced appropriately when heart disease is suspected. The dose should be adjusted according to TSH response and clinical conditions, such as adverse cardiac side effects. Some evidence reveals that subclinical hypothyroidism with serum TSH above 10 mIU/L requires no treatment in patients over 85 years old, because subclinical hypothyroidism in this subgroup does not lead to adverse events but will, instead, prolong life^20^, ^21^.

### Readmission, Anemia, and Blood Transfusion

The perioperative blood transfusion rate was defined as the number of patients having transfusion/the total number in respective cohort. Patients who were readmitted to the hospital for any reason during the 90 days after the first discharge were defined as hospital readmissions. The transfusion rate and hospital readmission were recorded and compared between the two cohorts. High transfusion and readmission rates mean increased blood loss from the TKA and increased risk of worse outcomes. Both parameters indicated the increased cost of this operation.

### Complications

The primary outcomes included the occurrence of complications within 90 days. Perioperative complications were classified into medical complications and surgical complications. Complications that occurred in the patient within 90 days postoperatively were tracked through ICD‐9 coding, recorded, and then compared.

#### Medical Complications

Medical complications included acute postoperative anemia, sepsis, intubation, pulmonary embolism, deep venous thrombosis (DVT), pneumonia, acute renal failure, cardiac complication, stroke, peripheral vascular complication, urinary tract infection, and pulmonary insufficiency. A particular complication would be confirmed by the specialist and positive laboratory and examination results. For example, if a patient experienced leg pain or swelling, ultrasonography would be arranged to exclude DVT. DVT was confirmed according to ultrasound findings and by specialists in vascular surgery. Having more medical complications resulted in poor outcomes and recovery from TKA.

#### Surgical Complications

Surgical complications included PJI and peri‐prosthetic fractures around the prosthetic joint. These complications were confirmed by the orthopaedists and positive laboratory and examination results. For patients with suspected PJI, laboratory results, including for routine blood tests, C‐reactive protein (CRP), erythrocyte sedimentation rate (ESR), interleukin‐6 (IL‐6), synovial leukocyte counts, and microorganisms collected from articular fluid, periprosthetic tissues and the prosthesis, were assessed and the diagnosis for this condition followed the American Academy of Orthopaedic Surgeons (AAOS) guidelines^22^. Pollution and iatrogenic infection were strictly avoided during the collection period. In addition, these patients must disuse any antibiotics for at least 2 weeks before the tests and examinations. Any peri‐prosthetic fractures around the prosthetic joint were confirmed by CT scans and X rays. More surgical complications were associated with increased incidence of reoperation and greater costs for patients and medical insurers.

### Statistical Analysis

The mean and the standard deviation or the median and the semi‐quartile range were used to describe continuous variables and compared using the two‐tailed *t*‐test. Ordinal variables were demonstrated by proportions and compared with the χ^2^‐test. A risk analysis model using logistic regression was conducted to estimate the odds ratios (OR) and 95% confidence intervals (CI) to identify risk factors for complications over a 90‐day period adjusted for preoperative and intraoperative variables. Multivariate logistic models were conducted with stepwise elimination of variables of interest from univariate analysis after adjustment for confounding factors. The statistical significance and power of analysis were *P*‐value ≤0.05 and 0.8, respectively. SPSS version 22 (Chicago, IL, USA) was used to perform all analyses.

## Results

### General Results

A total of 398 patients had a diagnosis of subclinical hypothyroidism. Patients were followed up for an average duration of 25.4 months (6 to 43 months). Among them, 275 cases (69.1%) were described as mild (79 patients [19.8%] with low FT4 and 196 patients [49.2%] with normal FT4 in the repeated test) and 123 cases (30.9%) as severe subclinical hypothyroidism. Of the 196 patients (49.2%) with mild subclinical hypothyroidism and normal FT4, 63 patients (15.8%) had symptoms before surgery. A total of 265 patients (66.6%) received preoperative treatment for subclinical hypothyroidism, with an average therapy time of 9.2 months. There were 162 patients (40.7%) with positive autoantibodies to thyroid peroxidase (anti‐TPO). Table 1 shows no statistically significant differences in baseline data between cohorts (all *P* > 0.05).

**TABLE 1:** Comparisons between baseline data with and without a diagnosis of subclinical hypothyroidism

Variable	Subclinical hypothyroid (*n* = 398)	Euthyroid (*n* = 398)	*P‐*value
Mean age ± SD (years)	64.8 ± 5.7	65.1 ± 5.4	0.446
Female (%)	351 (88.2)	349 (87.7)	0.337
Mean BMI ± SD (kg/m^2^)	25.5 ± 3.7	25.1 ± 3.5	0.118
Smoking history (%)	80 (20.1)	73 (18.3)	0.599
Heavy drinking (%)	42 (10.6)	38 (9.5)	0.724
Hypertension (%)	122 (30.7)	110 (27.6)	0.391
Cardiac diseases (%)	62 (15.6)	58 (14.6)	0.766
COPD (%)	21 (5.3)	17 (4.3)	0.618
Dialysis (%)	3 (0.8)	2 (0.5)	0.998
Diabetes mellitus (%)	87 (21.9)	92 (23.1)	0.734
Preoperative albumin <3.5 g/dL (%)	32 (8.0)	26 (6.5)	0.495
Diagnosis			
OA	343 (86.2)	352 (88.4)	0.575
RA	41 (10.3)	36 (9.0)	
Others	14 (3.7)	10 (2.5)	
ASA class			
I–II	388 (97.5)	385 (96.7)	0.672
Operative data			
Epidural anesthesia (%)	353 (88.7)	360 (90.5)	0.487
Mean operative time (min) (SD)	64.5 ± 7.2	63.7 ± 6.8	0.108
Perioperative blood transfusion rate (%)	78 (19.6)	60 (15.1)	0.030*
Length of stay (days)	13.6 ± 3.5	10.8 ± 3.8	<0.001*
Discharge disposition (%)			
Home	312 (78.4)	345 (86.7)	0.005*
Professional medical and nursing institutions after discharge	84 (21.1)	53 (13.3)	
Death	2 (0.5)	0 (0.0)	

ASA, American Society of Anaesthesiologists; BMI, body mass index; COPD, chronic obstructive pulmonary disease; OA, osteoarthritis; RA, rheumatoid arthritis; SD, standard deviation; TKA, total knee arthroplasty.

* Indicates statistical significance. Percentages are shown in parentheses.

### Readmission, Anemia, and Blood Transfusion

Subclinical hypothyroidism was associated with a greater incidence of readmission within 90 days after TKA compared to those without this condition (5.3% *vs* 1.5%, OR = 3.64, 95% CI = 3.46–3.82, *P* = 0.006). In addition, subclinical hypothyroidism was associated with greater odds of acute postoperative anemia (7.3% *vs* 4.3%, OR = 1.76, 95% CI = 1.67–1.85, *P* = 0.039) and requiring a blood transfusion (19.6% *vs* 15.1%, OR = 1.37, 95% CI = 1.30–1.44, *P* = 0.030) (see Table 1).

### Complications

Analysis of 90‐day cumulative results showed that patients with a diagnosis of subclinical hypothyroidism had an elevated risk of both medical and surgical complications postoperatively compared with those without this condition (11.6% *vs* 7.2%, OR = 1.55, 95% CI = 1.47–1.62, *P* < 0.05) (see Table 2).

**TABLE 2:** Comparison between the incidence of 90‐day postoperative complications among patients undergoing TKA with and without a diagnosis of subclinical hypothyroidism

Variable (*n*, %)	Subclinical hypothyroid (*n* = 398)	Euthyroid (*n* = 398)	OR	95% CI	*P*‐value
Medical complications					
Acute postoperative anemia	29 (7.3)	17 (4.3)	1.76	1.67–1.85	0.039*
Sepsis	12 (3.0)	3 (0.8)	4.10	3.80–4.31	0.037*
Intubation	2 (0.5)	1 (0.3)	2.00	1.90–2.10	0.998
Pulmonary embolism	4 (1.0)	0 (0)	4.12	3.83–4.32	0.180
Deep venous thrombosis	13 (3.3)	3 (0.8)	4.45	4.23–4.67	0.023*
Pneumonia	11 (2.8)	3 (0.8)	3.76	3.57–3.95	0.037*
Acute renal failure	4 (1.0)	1 (0.3)	4.03	3.83–4.23	0.370
Cardiac complication	14 (3.5)	4 (1.0)	3.59	3.41–3.77	0.032*
Stroke	15 (3.8)	3 (0.8)	5.16	4.90–5.42	0.009*
Peripheral vascular complication	2 (0.5)	0 (0)	2.00	1.90–2.10	0.998
Urinary tract infection	11 (2.8)	2 (0.5)	5.63	5.35–5.91	0.025*
Pulmonary insufficiency	12 (3.0)	3 (0.8)	4.10	3.80–4.31	0.037*
Surgical complications					
Periprosthetic infection	2 (0.5)	0 (0)	2.00	1.90–2.10	0.998
Peri‐prosthetic fracture around prosthetic joint	1 (0.3)	0 (0)	1.00	0.95–1.05	0.317
Other data					
Perioperative blood transfusion rate (%)	78 (19.6)	60 (15.1)	1.37	1.30–1.44	0.030*
Hospital readmission	21 (5.3)	6 (1.5)	3.64	3.46–3.82	0.006*

CI, confidence interval; OR, odds ratio; TKA, total knee arthroplasty.

* Indicates statistical significance. Percentages are shown in parentheses.

#### Medical Complications

Patients diagnosed with subclinical hypothyroidism had higher odds of multiple individual medical complications than those in the matched cohort. Specifically, postoperative infections, including sepsis (3.0% *vs* 0.8%, OR = 4.10, 95% CI = 3.80–4.31, *P* = 0.037), pneumonia (2.8% *vs* 0.8%, OR = 3.76, 95% CI = 3.57–3.95, *P* = 0.037), and urinary tract infections (2.8% *vs* 0.5%, OR = 5.63, 95% CI = 5.35–5.91, *P* = 0.025), were more common in patients with subclinical hypothyroidism (see Table 2).

Deep venous thrombosis (3.3% *vs* 0.8%, OR = 4.45, 95% CI = 4.23–4.67, *P* = 0.023), cardiac complications (3.5% *vs* 1.0%, OR = 3.59, 95% CI = 3.41–3.77, *P* = 0.032) and stroke (3.8% *vs* 0.8%, OR = 5.16, 95% CI = 4.90–5.42, *P* = 0.009) were more common in the case group than in the matched controls (see Table 2).

As for the incidence of intubation, pulmonary embolism, acute renal failure, and peripheral vascular complication, we failed to find a significant difference between the two groups (all *P* > 0.05, see Table 2).

#### Surgical Complications

There was no significant difference in PJI between the two cohorts (*P* > 0.05). As for the incidence of peri‐prosthetic fractures around the prosthetic joint, we also failed to find a significant difference between the two groups (all *P* > 0.05, see Table 2).

### Logistic Regression Analysis for 90‐Day Complications Following Total Knee Arthroplasty

Controlling for preoperative and intraoperative variables, patients with TSH ≥ 10 mu/L (OR = 7.34, 95% CI = 7.01–7.19, *P* < 0.001), with positive anti‐TPO (OR = 6.23, 95% CI = 5.92–6.26, *P* < 0.001), without corrected subclinical hypothyroid (OR = 6.21, 95% CI = 5.90–6.52, *P* < 0.001), and without thyroid hormone supplementation (OR = 3.65, 95% CI = 3.47–3.83, *P* = 0.012) were more likely to experience postoperative complications within 90 days of surgery (Table 3).

**TABLE 3:** Multivariate analysis of risk factors for 90‐day postoperative complications after TKA

Risk factor	OR	95% CI^*^	*P*‐value
TSH ≥ 10 mu/L	7.34	7.01–7.19	<0.001^†^
Without corrected subclinical hypothyroid	6.21	5.90–6.52	<0.001^†^
Without thyroid hormone supplementation	3.65	3.47–3.83	0.012^†^
With hypothyroid symptoms	2.25	2.00–2.23	0.432
Anti‐TG (+)	1.25	1.56–1.72	0.762
Anti‐TPO (+)	6.23	5.92–6.26	<0.001^†^

CI, confidence interval; OR, odds ratio; TG, thyroglobulin; TKA, total knee arthroplasty; TPO, thyroid peroxidase

TSH, thyroid stimulating hormone

* Fully adjusted by confounding variables. Odds ratios, as well as 95% confidence intervals, are shown. Percentages are shown in parentheses.

^†^ Indicates statistical significance.

### Subgroup Analysis

Table 4 presents details of the the subgroup analysis for mild (*N* = 275) and severe (*N* = 123) subclinical hypothyroidism. Severe subclinical hypothyroidism was associated with an increased incidence of readmission within 90 days after TKA (2.5% *vs* 11.4%, OR = 4.89, 95% CI = 4.65–5.13, *P* = 0.001) compared to mild subclinical hypothyroidism. However, there were no significant differences in the incidence of acute postoperative anemia (6.9% *vs* 7.3%, OR = 1.06, 95% CI = 1.01–1.11, *P* = 0.948) and blood transfusion (20.4% *vs* 17.9%, OR = 0.87, 95% CI = 0.83–0.91, *P* = 0.661) between groups. In addition, those with severe subclinical hypothyroidism had higher odds of experiencing postoperative infections, including sepsis (1.5% *vs* 6.5%, OR = 4.43, 95% CI = 4.21–4.65, *P* = 0.016), pneumonia (1.5% *vs* 5.7%, OR = 3.91, 95% CI = 3.71–4.11, *P* = 0.040), and urinary tract infections (1.1% *vs* 6.5%, OR = 6.12, 95% CI = 5.81–6.43, *P* = 0.007) (see Table 4) compared to those with mild subclinical hypothyroidism. As for the rest of the complications, there were no significant differences between groups (all *P* > 0.05, see Table 4).

**TABLE 4:** Subgroup analysis for mild (*N* = 275) and severe (*N* = 123) subclinical hypothyroidism

Variable (*n*, %)	Mild subclinical hypothyroid (*n* = 275)	Severe subclinical hypothyroid (*n* = 123)	OR^†^	95% CI	*P‐*value
Medical complications					
Acute postoperative anemia	19 (6.9)	9 (7.3)	1.06	1.01–1.11	0.948
Sepsis	4 (1.5)	8 (6.5)	4.43	4.21–4.65	0.016*
Intubation	1 (0.4)	1 (0.8)	2.11	0.97–1.07	0.856
Pulmonary embolism	2 (0.7)	2 (1.6)	2.35	2.00–2.47	0.774
Deep venous thrombosis	8 (2.9)	5 (4.1)	1.55	1.47–1.63	0.769
Pneumonia	4 (1.5)	7 (5.7)	3.91	3.71–4.11	0.040*
Acute renal failure	2 (0.7)	2 (1.6)	2.35	2.23–2.47	0.774
Cardiac complication	8 (2.9)	6 (4.8)	1.72	1.63–1.81	0.490
Stroke	10 (3.6)	5 (4.1)	1.21	1.15–1.27	0.938
Peripheral vascular complication	1 (0.4)	1 (0.8)	2.20	2.09–2.31	0.856
Urinary tract infection	3 (1.1)	8 (6.5)	6.12	5.81–6.43	0.007*
Pulmonary insufficiency	8 (2.9)	4 (3.3)	1.51	1.43–1.59	0.895
Surgical complications					
Periprosthetic infection	0 (0)	2 (1.6)	1.70	1.62–1.78	0.176
Peri‐prosthetic fracture around prosthetic joint	1 (0.4)	0 (0)	0.89	0.85–0.94	0.679
Other data					
Perioperative blood transfusion rate (%)	56 (20.4)	22 (17.9)	0.87	0.83–0.91	0.661
Hospital readmission	7 (2.5)	14 (11.4)	4.89	4.65–5.13	0.001*

CI, confidence interval; OR, odds ratio; TSH, thyroid stimulating hormone

* Indicates statistical significance

^†^ Fully adjusted by confounding variables. Odds ratios, as well as 95% confidence intervals, are shown. Percentages are shown in parentheses.

Table 5 presents details of the subgroup analysis for low FT4 and normal FT4 in mild and severe subclinical hypothyroidism cases. In the mild subclinical hypothyroidism group, there was no significant difference for all the complications (all *P* > 0.05). However, in the severe subclinical hypothyroidism group, there was increased incidence of postoperative infections, including sepsis (24.1% *vs* 1.1%, *P* < 0.001), pneumonia (20.7% *vs* 1.1%, *P* < 0.001), urinary tract infection (20.7% *vs* 2.1%, *P* = 0.002), and hospital readmission (41.4% *vs* 2.1%, *P* < 0.001) in the low FT4 compared to the normal FT4 subgroup.

**TABLE 5:** Subgroup analysis for low FT4 and normal FT4 subclinical hypothyroidism

Variable (*n*, %)	Mild subclinical hypothyroid (n = 275)			Severe subclinical hypothyroid (*n* = 123)		
	Low FT4 (*n* = 79)	Normal FT4 (*n* = 196)	*P*‐value	Low FT4 (*n* = 29)	Normal FT4 (*n* = 94)	*P*‐value
Medical complications						
Acute postoperative anemia	7 (8.9)	12 (6.1)	0.584	3 (10.3)	6 (6.4)	0.758
Sepsis	2 (2.5)	2 (1.0)	0.696	7 (24.1)	1 (1.1)	<0.001*
Intubation	0 (0.0)	1 (0.5)	0.638	1 (3.4)	0 (0.0)	0.532
Pulmonary embolism	1 (1.3)	1 (0.5)	0.907	1 (3.4)	1 (1.1)	0.962
Deep venous thrombosis	3 (3.8)	5 (2.6)	0.873	3 (10.3)	2 (2.1)	0.155
Pneumonia	2 (2.5)	2 (1.0)	0.696	6 (20.7)	1 (1.1)	<0.001*
Acute renal failure	1 (1.3)	1 (0.5)	0.907	1 (3.4)	1 (1.1)	0.962
Cardiac complication	5 (6.3)	3 (1.5)	0.081	2 (6.9)	4 (4.3)	0.933
Stroke	4 (5.1)	6 (3.1)	0.655	2 (6.9)	3 (3.2)	0.730
Peripheral vascular complication	1 (1.3)	0 (0.0)	0.638	1 (3.4)	0 (0.0)	0.532
Urinary tract infection	2 (2.5)	1 (0.5)	0.413	6 (20.7)	2 (2.1)	0.002*
Pulmonary insufficiency	5 (6.3)	3 (1.5)	0.081	2 (6.9)	2 (2.1)	0.505
Surgical complications						
Periprosthetic infection	0 (0)	0 (0.0)	‐	1 (3.4)	1 (1.1)	0.962
Peri‐prosthetic fracture around prosthetic joint	1 (1.3)	0 (0.0)	0.638	0 (0)	0 (0.0)	‐
Other data						
Perioperative blood transfusion rate (%)	17 (21.5)	39 (19.9)	0.891	7 (24.1)	15 (16.0)	0.467
Hospital readmission	2 (2.5)	5 (2.6)	0.679	12 (41.4)	2 (2.1)	<0.001*

FT4, serum‐free thyroxine

* Indicates statistical significance. Percentages are shown in parentheses.

## Discussion

Despite the high prevalence of subclinical hypothyroidism in the elderly^7^, ^8^, there is limited research on its impact on postoperative results. The present study revealed that subclinical hypothyroidism was a risk factor for 90‐day medical and surgical complications after TKA. This finding might arise from the direct and indirect influences of thyroid hormones on platelet maturation and function, the synthesis and action of coagulation factors, and the maintenance of blood viscosity^23^.

### Ninety‐Day Postoperative Complications

The results of this study concur with the results on the relationship between hypothyroidism and PJI in previous studies^9^, ^10^. Despite there not being a statistically significant difference in the present sample population, all patients with PJI (*n* = 2) had subclinical hypothyroidism. This finding suggests that subclinical hypothyroidism also has connections with an elevated risk of postoperative PJI in comparison with those without this condition, similar to the increased odds of PJI in hypothyroidism reported by Tan *et al*. and Leonard *et al*.^9^, ^10^ The increased risk of infectious processes may be associated with the weakening role of the thyroid hormone in modulating the cell‐mediated immunity^24^. These roles of thyroid hormones could help explain why subclinical hypothyroidism patients experienced significantly higher incidences of sepsis (OR = 4.10), pneumonia (OR = 3.76), and urinary tract infections (OR = 5.63) in comparison with the matched controls.

### Readmission

Patients in the present study with subclinical hypothyroidism experienced more unplanned hospital readmissions and had to seek professional medical help after discharge, and, therefore, incurred further medical costs. Higher complication rates in subclinical hypothyroidism patients would be another main contributor to higher costs when compared to matched controls. These findings could explain the concern expressed by 94% of members the American Association of Hip and Knee Surgeons of the financial disincentive of selecting high‐risk patients as candidates for arthroplasty^25^. Therefore, screening for, monitoring, and treating underlying metabolic abnormalities, previously regarded as “mild” thyroid disease, may not only improve outcomes following primary TKA but also reduce its economic burden on individuals and medical insurers.

### Which Subclinical Hypothyroidism Patients Suffered Increased Risks for 90‐Day Postoperative Complications

The effects of thyroid dysfunction on the clinical outcomes following TKA are various. In fact, thyroid dysfunction involves different conditions, including hyperthyroidism, hypothyroidism, subclinical hyperthyroidism, and corrected hyperthyroidism. Following on from previous studies demonstrating that hyperthyroidism and hypothyroidism could complicate surgical procedures and compromise recovery from TKA^9^, ^19^, ^20^, ^22^, the present findings added the possibility that other thyroid dysfunctions also influence postoperative results. The American Association of Clinical Endocrinologists, the US Endocrine Society, and the American Thyroid Association convened a panel that found evidence that subclinical hypothyroidism has a tendency towards overt hypothyroidism^17^. Subclinical hypothyroidism progresses to overt hypothyroidism in approximately 2%–5% of hypothyroidism cases annually^26^. Specifically, patients with subclinical hypothyroidism, especially those with TSH ≥ 10 mu/L, without corrected subclinical hypothyroidism, without thyroid hormone supplementation, and with positive anti‐TPO, could suffer greater risk of adverse events after TKA. It is worth noting that previous studies have shown that the detection of thyroid peroxidase antibody (anti‐TPO) is useful in patients with subclinical hypothyroidism because the presence of anti‐TPO confirms that autoimmunity is the cause of subclinical hypothyroidism. Anti‐TPO also plays a role in predicting progression to overt hypothyroidism^17^, ^18^.

Clinical associations with isolated TSH elevations may not relate to mild thyroid gland dysfunction but may reflect the severity of nonthyroidal illness or other factors. Isolated TSH elevations in chronically ill patients were associated with impaired cardiac function in a small cross‐sectional study^27^, with higher mortality risk in a retrospective study^28^. In prospective cohort study of chronically ill patients, isolated TSH increases between 10 and 20 mU/L were associated with increased incidence and recurrence of heart adverse events^29^. In a large retrospective study of patients with chronic heart failure, TSH levels >4.6 mU/L were associated with increased mortality risk^30^. The candidates for TKA are generally elderly, and, hence, it is hard for them to avoid chronic diseases. These could help partly explain why they suffered more risks in the present cohort.

### Subgroup Analysis for Mild and Severe Subclinical Hypothyroidism

Subclinical hypothyroidism was classified as mild (TSH < 10 mu/L) or severe (TSH ≥ 10 mu/L)^11^. Our results demonstrated that the level of serum TSH is only associated with the complications involving infections, such as sepsis, pneumonia, and urinary tract infections. Namely, the levels of subclinical hypothyroidism have a close connection with the occurrence of infection. This possibly arises from the crucial role of thyroid hormones in modulating the cell‐mediated immunity^24^. Postoperative infection compromises the quality of life of patients and increases the economic burden on both individuals and medical insurers. Thus, the levels of TSH could be a helpful indicator for poor prognosis and increased costs after TKA.

### Implications

At present, there is no recommendation for routine screening for thyroidal dysfunction in patients who have no history of this condition before TKA. Considering that the candidates for TKA are generally elderly and this condition overlaps with subclinical hyperthyroidism, it is necessary for orthopaedists to perform a preoperative TSH assessment, especially for those aged >65 years, rather than only screening individuals with suspected thyroid disease or with known hypothyroidism (or hyperthyroidism), so that treatment can be given prior to TKA. Surgeons should postpone TKA, as an elective surgery for some patients with subclinical hypothyroidism, such as those with TSH ≥ 10 mu/L and positive anti‐TPO, until appropriate treatments and adequate care can allow thyroid hormones to achieve euthyroidism.

### Limitations

Several limitations exist in the present case‐control study. First, the small sample size in our study could lead to a failure to detect a statistically significant difference in the odds of comorbid conditions and complications such as PJI, intubation, pulmonary embolism, and acute renal failure. Second, the study design aimed to investigate the short‐term complications following TKA in patients with subclinical hypothyroidism. At present, the follow‐up period for these patients is not long enough to draw a conclusion regarding whether the subclinical hypothyroidism remains to increase long‐term complications. Third, currently, in chronically ill patients, there is inadequate evidence to determine: (i) that isolated TSH elevations usually persist or progress to overt hypothyroidism; (ii) the etiology and clinical significance of isolated TSH elevations; and (iii) whether levothyroxine therapy is indicated for persistent isolated TSH elevations^31^. Thus, there exists poor homogeneity for the diagnosis and treatments of subclinical hypothyroidism in our cohorts. Fourth, the statistical methodology used in our study connected subclinical hypothyroidism with elevated complications following primary TKA. These results do not show a strong causal relationship between subclinical hypothyroidism and the observed complications because this study did not completely control potential confounders affecting the outcomes we are interested in, such as recall bias from the collected medical history. Finally, we did not observe a difference in the incidence of outcomes of hypothyroid versus corrected versus euthyroid comparisons, and future studies examining this would provide greater confidence for our conclusions in the present study.

### Conclusion

The present study suggested that subclinical hypothyroidism could increase the risk of postoperative complications within 90 days of TKA, especially for patients with TSH ≥ 10 mu/L and positive anti‐TPO and those without corrected subclinical hypothyroid and thyroid hormone supplementation.
